# Wireless Distributed Environmental Sensor Networks for Air Pollution Measurement—The Promise and the Current Reality

**DOI:** 10.3390/s17102263

**Published:** 2017-10-02

**Authors:** David M. Broday, the Citi-Sense Project Collaborators

**Affiliations:** 1 Faculty of Civil and Environmental Engineering, Technion IIT, 32000 Haifa, Israel

**Keywords:** wireless distributed environmental sensor networks, micro sensing units, air pollution, in situ field calibration, spatiotemporal variability, multi-sensor nodes

## Abstract

The evaluation of the effects of air pollution on public health and human-wellbeing requires reliable data. Standard air quality monitoring stations provide accurate measurements of airborne pollutant levels, but, due to their sparse distribution, they cannot capture accurately the spatial variability of air pollutant concentrations within cities. Dedicated in-depth field campaigns have dense spatial coverage of the measurements but are held for relatively short time periods. Hence, their representativeness is limited. Moreover, the oftentimes integrated measurements represent time-averaged records. Recent advances in communication and sensor technologies enable the deployment of dense grids of Wireless Distributed Environmental Sensor Networks for air quality monitoring, yet their capability to capture urban-scale spatiotemporal pollutant patterns has not been thoroughly examined to date. Here, we summarize our studies on the practicalities of using data streams from sensor nodes for air quality measurement and the required methods to tune the results to different stakeholders and applications. We summarize the results from eight cities across Europe, five sensor technologies-three stationary (with one tested also while moving) and two personal sensor platforms, and eight ambient pollutants. Overall, few sensors showed an exceptional and consistent performance, which can shed light on the fine spatiotemporal urban variability of pollutant concentrations. Stationary sensor nodes were more reliable than personal nodes. In general, the sensor measurements tend to suffer from the interference of various environmental factors and require frequent calibrations. This calls for the development of suitable field calibration procedures, and several such in situ field calibrations are presented.

## 1. Introduction

Recent developments in sensory and communication technologies have made the deployment of small, portable, and relatively low-cost Monitoring Sensor Units (MSUs) possible [1]. These MSUs operate as a set of standalone nodes, with each node housing several sensors for different ambient pollutants and some meteorological parameters. The deployed nodes communicate with a central data storage and management server through a hierarchical architecture, thus forming a Wireless Distributed Environmental Sensor Network (WDESN). The deployment of numerous nodes enables us to gather highly resolved spatial and temporal data [2], therefore allowing for a better smoothing of the discrete measurements via interpolation [3,4] and for developing regression [5] or pollutant dispersion [6,7] models. As such, data from WDESNs are expected to result in closer to real-life pollution patterns [8]. The sensors for the measurement of gaseous pollutants belong to one of the following technologies: amperometric (metal oxide—MO) sensors [9], electronic circuitry (electrochemical—EC) sensors [10], and non-dispersive IR (NDIR) sensors [11]. The sensors for measuring the concentration of airborne particles include capacitive solid state sensors [12], miniaturized optical particles counters (OPCs), detectors of radiation absorption, or particle spectrometers [13,14]. Depending on the sensor technology and algorithm, these sensors enable us to measure the particle number concentration (PNC) or the particulate mass concentration (particulate matter—PM). The emergence of these relatively low-cost sensor technologies opened new applications for air pollution data gathering beyond the regulatory and scientific uses. Such applications include the empowerment of citizens by providing them with quantitative information about pollutant levels in their vicinity, facilitating the measurement of indoor air quality at home and/or in public spaces, enabling measurement by mobile/personal/wearable sensors rather than only by stationary high-end instruments, etc. Theoretically, static nodes can provide continuous measurements over long times, i.e., high temporal resolution over long periods at each location. In contrast, mobile nodes can provide better spatial coverage of the study area at the expense of lower temporal resolution in each location [2,15]. Yet both claims need to be scrutinized.

Due to the sensors’ lower-quality, WDESN nodes require frequent calibrations. Laboratory calibration of the individual sensors is performed in a controlled atmosphere (temperature, relative humidity, still air, mostly one pollutant at a time, etc.) [16]. This setup is very different from the conditions the sensors actually experience when they are deployed in urban environments. For example, the effects of the sensor velocity or of wind gusts on its performance may be substantial [17]. Similarly, the vibrations of mobile MSUs while the measurement is performed can result in artifacts [18]. These factors suggest that stationary WDESN nodes may provide more reliable data than mobile nodes. Moreover, stationary measurement opens different, easier, and more versatile options for sensor calibration.

In a previous study [19], we examined MSUs that contained metal oxide (MO) chemoresistive sensors for O_3_, NO_2_, and total volatile organic compounds (TVOC), testing their suitability for measuring ambient pollutant levels and for capturing their spatiotemporal variability. We proposed a few field calibration procedures and applied them for O_3_ sensor measurements. However, these calibration procedures can be applied only when the pollutant concentrations exhibit negligible spatial variability for a sufficiently long time-period in which the reported concentrations are above the sensors’ quantification level. Unfortunately, these criteria do not always hold for, e.g., NO_2_ in urban areas. In this work, after briefly presenting these concepts, we discuss further results and additional ambient pollutants. The measurements were obtained by different sensor technologies and WDESN platforms, both stationary and personal.

## 2. Materials and Methods

### 2.1. Static Platforms

The battery operated static MSUs (AQMesh v3.5, Environmental Instruments Ltd., Burton-on-Trent, UK) contained four electrochemical sensors (AlphaSense, Essex, UK) for gaseous pollutants: CO, NO, NO_2_, and O_3_ (Table 1), with the NO_2_ sensor re-designed to reject O_3_ and eliminate its cross-sensitivity. In addition, the MSU contained a particle number concentration (PNC) sensor that measured 32 equally spaced size channels (Table 2). Particulate matter (PM), i.e., mass concentrations of particles smaller than 10 μm in diameter (PM_10_) and of particles smaller than 2.5 μm in diameter (PM_2.5_), was reported by a proprietary algorithm (run by the MSU manufacturer) that converted the size-specific particle counts into PM, assuming spherical particle shape and standard density. Moreover, a set of proprietary algorithms has been run by the platform manufacturer for the post-processing of the observed sensor records, aiming to correct for cross-interference and for the effects of temperature and relative humidity. In parallel, AC-powered static MSUs (Elm, Perkin Elmer, Waltham, MA, USA) that contained three metal oxide (MO) chemoresistive sensors for O_3_, NO_2_, and TVOC were also used (Table 1). Reference ambient pollutant concentrations (30 min resolution) were obtained from the Neve Shaanan air quality monitoring (AQM) station, which is situated in the neighborhood that served as our study area. Battery/AC powered mini optical particle counters (OPCs) (Dylos 1700 DC, Dylos Co., Riverside, CA, USA) were used for the PNC measurements.

### 2.2. Mobile Platform

Battery operated Little Environmental Observatory (LEO) nodes (Ateknea, Barcelona, Spain) were used as personal MSUs and contained EC sensors for three gaseous pollutants: CO, NO_2_, and O_3_ (Table 3). The nodes could communicate with the user’s smartphone via Bluetooth and a proprietary application but were set to operate in a detached (stand-alone) mode. It is noteworthy that, whereas both the LEO (personal) and the AQMesh (stationary) nodes used electrochemical sensors, the algorithm ran by the LEOs (unlike the one ran by the AQMesh) did not correct for ambient temperature and relative humidity variations, which have a critical role on the sensors’ performance [10]. Hence, based on data provided to us by the manufacturer, we implemented a correction for the temperature effect on the zero-current, which affects the net current of the working electrode in EC cells. It should be noted that the difference between the A-type (Table 3) and the B-type (Table 1) Alphasense sensors is merely the sensor package and size and that the sensors have similar characteristics with respect to cross interference and sensitivity to temperature and humidity. The personal PNC measurements were performed by the battery operated Tzoa-RD (Tzoa, Vancouver, British Columbia, Canada), which was set according to the manufacturer guidelines. The nodes could communicate with the user smartphone via Bluetooth and a proprietary application but were set to operate in a stand-alone mode.

### 2.3. Sensor Evaluation

#### 2.3.1. Laboratory Experiments

The performance of the sensors against traceable gas standards was evaluated at atmospheric pressure under controlled conditions (temperature between 20 to 30 ± 0.5 °C and relative humidity between 30 to 70 ± 1%). O_3_ was produced using a UV lamp generator, and NO_2_ was generated by gas phase titration of O_3_ and NO. A standard dilution system with zero-air was used for varying the concentrations. The measurements were performed by pre-calibrated Teledyne analyzers (Table 4). Measurements by the sensor nodes of individual gaseous pollutants provided information on the sensors’ cross-sensitivity.

#### 2.3.2. Field Experiments

The evaluation of the WDESN sensors in the field was done in several ways, both while the MSUs were collocated at an AQM station and while they were fully deployed (either in pairs or individually) in a typical urban area. The specific details varied from campaign to campaign and, for clarity, will be described when the results of the specific campaigns are reported. Briefly, in a previous field study [19], we reported poor performance, limited selectivity (specificity), and degradation of MO sensors for gaseous pollutants. The testing of the capabilities of EC sensors for gaseous pollutants along the same lines (i.e., in a field campaign) is reported here. Clearly, different performance criteria may be suitable for different applications (i.e., regulatory, scientific, utilization by NGOs, personnel use, citizen empowerment, etc.), allowing task-specific MSU performance assessment. For this, a comprehensive Sensor Evaluation Toolkit (SET) was developed [20], which enables one to evaluate the performance of sensor nodes and to compare them using different metrics. Initially, the SET was used to evaluate 25 sensors that were collocated at AQM stations in eight cities in Europe (Barcelona, Belgrade, Edinburgh, Haifa, Ljubljana, Oslo, Ostrava, Vienna) [20] as part of the EU FP7 Citi-Sense project. The evaluation involved multiple comparisons of paired observation time-series: one acquired by the MSU and one measured by the collocated reference AQM device. An inter-comparison of the pollutant-specific SMU sensors to each other was also performed. The SET reports several performance measures, including the overall root mean square error (RMSE), Pearson correlation, Spearman correlation, Kandall correlation, the completeness of the sensor data (termed presence), the sensor accuracy at varying temporal scales (termed match), the sensor capability to capture the temporal variability in the reference AQM data (termed Lower Frequencies Energy Content, LFE), and a combined metric, the Integrated Performance Index (IPI).

## 3. Results

### 3.1. Laboratory Evaluation

Table 5 presents the performance of different EC sensors in the lab. For all the pollutants, the sensors showed good correlations with the reference instruments (*r* > 0.9), revealing low (O_3_) or no (NO_2_, NO_2_) cross-sensitivity. In particular, the new NO_2_ sensor (Table 1) addressed the cross-sensitivity to O_3_ that the preceding EC NO_2_ sensor [16] showed by effectively rejecting O_3_. Yet, in general, the sensors were not accurate and showed pollutant-specific bias. After calibration, the sensors provided reliable measurements at the laboratory under steady temperature and relative humidity conditions. However, it is noteworthy that such scenarios do not represent common ambient conditions.

### 3.2. Field Evaluation 

#### 3.2.1. Stationary Nodes

Table 6 depicts the SET IPI values for several pollutants in 25 European cities, revealing that the MSUs show pollutant-specific IPIs that vary by geographical area and time of the year (which will be disentangled later). Table 7 presents the SET results for the PM_10_-transformed PNC data while the MSUs were collocated at an AQM station in Ostrava (CZ). The sensor performance varied but was generally acceptable, indicating reliable measurements. Similar results were obtained in the other cities, suggesting that the MSU PM measurements were fairly accurate. The high LFE value indicates that the spectral information content in the reference signal could be captured by the sampling rate of the sensors. Namely, the temporal patterns of the environmental phenomena could be discerned by the MSU measurements.

A similar breakdown of the SET IPI results for the data of Table 6 revealed that, whereas the NO sensors showed low correlations to the reference AQM data, their match scores were relatively high. Namely, while the EC NO sensors demonstrated poor performance to be considered suitable for regulatory applications (Pearson correlation <0.3, relatively large RMSE), they could still be useful for citizen science and educational purposes, where relative spatial patterns may be sufficient, in agreement with [21]. In fact, a careful inspection of the IPI components can provide quantitative support for decisions about the suitability of MSUs for specific applications/stakeholders.

#### 3.2.2. Mobile Nodes

The small size and low power-consumption of the stationary MSUs enabled us to test them also as mobile nodes, demonstrating the option to cover wide area with a small number of nodes. While there are many advantages for such a WDESN mode of operation [22], one cannot disregard the effect of the sensor’s motion on the measurement. Sampling during motion by AQM reference instruments [23] was shown to affect PM_2.5_ measurements due to non-isokinetic sampling. However, gaseous pollutant measurements were not affected, since the reference instruments operate at regulated flow rates. In contrast, the diffusion-based WDESN gaseous pollutant sensors are expected to be affected by mobile measurement. Specifically, airflow (either wind or due to the MSU motion) enhances the heat loss at the sensor face, which affects the behavior of the MSU passive sensors [24] since MO and EC sensors require temperature stability for reliable measurement (Section 3.1). Temperature variations, e.g., due to increased convective heat transfer, affect a the relative humidity at the sensor face, which was also found to alter the sensor readings [25]. The effect of motion on the WDESN sensor measurement was studied by placing MSUs in a wind tunnel and by attaching MSUs to vehicle roofs [17]. In both cases, the MSUs were set in different orientations relative to the airflow (facing forward, backward, or sideward). The wind tunnel experiments revealed that air speed had a clear effect on the measurement, with MO sensors reporting lower NO_2_ and higher O_3_ concentrations for increasing air velocities. The measurement artefact was reversible and vanished about 20 to 30 min after the air velocity ceased. The MSU orientation with respect to the airflow had a negligible effect on the measurement. In the field experiments (i.e., with the MSUs attached to vehicles), the measurement of the MSU temperature sensor was clearly affected by the vehicle speed. The readings of the O_3_ and NO_2_ sensors were also affected by the vehicle speed but only when the sensor was facing the direction of travel. For example, relative to stationary MSU measurements, sensors that faced the travel direction showed four to 15 fold lower NO_2_ concentrations and 1.5 to six fold higher O_3_ concentrations when the vehicles moved at speeds that ranged between 0 and 60 km/h [17].

#### 3.2.3. Personal Nodes

We evaluated the performance of nine collocated personal LEO MSUs (Table 3) against each other, as well as against AQM reference data. In general, the personal MSUs did not perform as well as the stationary MSUs, even after the implementation of temperature correction (Section 2.2), with the measurements showing high sensitivity to high RH conditions. Specifically, considerable increases in NO and NO_2_ measurements and decreases in O_3_ measurements were observed when the ambient RH sharply increased. Node-specific and pollutant-specific linear regressions of sensor measurements against AQM data revealed coefficients of determination (R^2^) that varied considerably among experiments (Table 8), with better performance of the NO_2_ sensors than of the other sensors. The inter-nodal correlations were mostly higher than the correlations with the AQM data.

We used the SET to evaluate the personal OPC (Tzoa-RD, Table 3) against the stationary (AQMesh, Table 2) OPC. We focused on PNC (i.e., before the MSU measurements were converted into PM), using data collected in Barcelona in Spring, 2016. The stationary OPC performed much better than the personal OPC by all but one IPI parameter. However, the higher sampling rate of the personal sensor (*f* = 1 min) relative to the stationary sensor (*f* = 15–60 min) resulted in richer data that led to a higher match score for the personal nodes.

These results suggest that reporting the Community Air Quality Index (CAQI) based on the highly temporally resolved sensor data is possible, may be favored by concerned citizens, and can also be used for educational purposes. Yet, as found for the stationary MSUs, personal sensor data are not suitable for scientific or regulatory applications that require accurate measurements. In fact, even for social applications, we recommend to average the 1 min sample data over 5 min or even 15 min (thus affecting the immediacy of data updates) to reduce the random measurement noise.

### 3.3. Effect of Meteorological Conditions on MSU Performance

Ambient conditions were shown to have a major effect on the sensors’ performance [16,17,19,20,26,27,28,29]. Due to the variability of the meteorological and climatological conditions among the eight Citi-Sense project cities (Table 6), we could examine how the measurement bias varied with the ambient temperature and relative humidity. In general, the meteorological conditions considerably affected the sensors’ performance, with the measurements varying even among sensors from the same batch [27]. The ambient pollutant levels also affected the sensor measurement, with very low pollutant levels resulting in low IPI scores and high measurement variability [19,20]. Higher ambient concentrations of primary pollutants are common in the winter [30] due to a thinner planetary boundary layer (i.e., reduced vertical mixing), lower photolysis rates of primary pollutants and consequently lower formation rates of secondary pollutants, and low-temperature-related emissions, e.g., biomass burning for space heating. Hence, considerable seasonal variability of MSU performance could be expected since the sensors have relatively high quantification limits (e.g., 30 ppb was suggested as the in situ quantification limit of NO_2_ and NO EC sensors [20], whereas lab tests of the same sensors suggested that it was 10 ppb). Thus, the more reliable data reported by WDESN nodes when they experience high pollutant levels result in seasonal variability in their performance.

### 3.4. Land Use Effects

The performance of WDESN MSUs was assessed while the nodes were deployed in different urban microenvironments. For example, in Haifa (IL), six MSUs (MO sensor technology) were deployed in pairs at three different sites, some 100 to 150 m apart, for 71 days in Spring-Summer of 2013 [19]. While collocated at the neighborhood AQM station, the correlations between the MSU measurements and the reference AQM data were 0.92 to 0.99 for O_3_, 0.77 to 0.99 for TVOC, and 0.78 to 0.98 for NO_2_. Lower correlations were obtained between the MSU measurements and the records from more distant AQM stations, indicating that the MSU observations were sensitive to their local environmental conditions. Next, the MSUs were deployed in the neighborhood, enabling one to study the sensor response to inner-neighborhood concentration variability. Specifically, upon MSU relocation (one of each pair at a time), the NO_2_ sensors adjusted very quickly to their new microenvironments and reported matched site-specific diurnal patterns. The diurnal patterns that were reported by the TVOC sensors that were deployed near a busy road were very similar to those of the NO_2_ sensors, suggesting that the TVOC sensors were sensitive to traffic-related pollution. The reported range of urban CO concentrations [31] suggested that the TVOC observations were probably mostly due to ambient CO (see Discussion for further details).

Moreover, in a different campaign, single MSUs (EC sensors) were collocated for 83 days in the Summer-Fall of 2015 at three AQM stations in Oslo (NO); two nodes near busy roads and one node in a calm street [27]. Intermediate NO_2_ correlations (<0.7) and high NO correlations were obtained in the three sites, with lower correlations (NO_2_: *r* = 0.5, NO: *r* = 0.8) at the AQM station in the low-traffic area and higher correlations at the AQM stations in the high-traffic areas (NO: *r* = 0.8–0.9). Similar results were obtained in Belgrade, with MSUs collocated at a traffic-affected AQM station (for 75 days in the Spring-Summer of 2015) and at an urban-background AQM station (for 94 days in the Summer–Fall of 2015). In contrast, for PM, higher correlations were obtained by the MSU that was collocated at the low-traffic area AQM (PM_10_: *r* = 0.7–0.8, PM_2.5_: 0.8–0.9) than by the MSUs that were collocated at the high-traffic area AQM (*r* < 0.4 for both PM fractions) [27]. The opposing performance of the MSU PM and gaseous sensors have two significant implications. First, the deployment plan of multi-sensor nodes may involve contradicting aspects. Namely, if the target is gaseous pollutants (in particular NO_x_), we showed that MSUs should be deployed near traffic arteries. In contrast, if PM is the target pollutant, then the nodes should be deployed away from busy traffic arteries, since the PM count-to-mass converted data are more reliable away from busy roads, where particles tend to be larger. Second, it suggests that exposure to ultrafine (UF) particles, which are believed to either be directly emitted by vehicles or to form from emissions of gaseous precursors, cannot be reliably assessed using the MSU PM data, which is ‘blind’ to UF particles. In contrast, the spatial variability of urban-scale PNC has been revealed by high-end instruments [32] as well as by low-end (Dylos) MSUs [33]. Since UF particles differ considerably from the larger particles found in background sites, due to aging and restructuring [34], and as they may have different toxicity, they inflict distinct adverse health effects [35]. Due to the high correlation between PNC and NO_x_ concentrations [36], if PNC is the target pollutant, the MSU deployment scheme should probably follow that of the gaseous pollutants.

To summarize, since the calibration coefficients supplied by the sensor/platform manufacturers are obtained in controlled exposure chambers and therefore do not provide reliable concentrations under real-world conditions, the post-processing of MSU observations is necessary. Based on multiple field experiments, we conclude that frequent in situ calibration of the MSUs under varying environmental conditions seems to be the best approach to account for the impact of site-specific varying environmental conditions on the MSU performance. Next, we describe several such in situ field calibration procedures.

### 3.5. Field Calibration

As indicated above, the intercept (offset) and slope (gain) obtained in laboratory calibration were different from those obtained in the field. For example, a CO EC sensor with an offset of 0.07 ppb in the lab had an offset of 166 ppb in the field [27]. This demonstrates that, without accounting for real environmental conditions, the performance of deployed MSUs could be poorer than the nominal sensor specifications, up to a point at which their data are completely unreliable. The simplest and most common approach for field calibration is collocation, referring to MSU measurements that are performed adjacent to a reference and periodically calibrated AQM analyzer. The SET can be used for evaluating the sensor performance before and after the calibration. For example, O_3_ observations in Haifa (IL) were highly correlated among collocated nodes but suffered from considerable inter-nodal bias [19]. Applying calibration (linear regression) while in collocation with an AQM station, the MSU-specific calibration coefficients of the O_3_ sensors significantly improved the overall WDESN performance, with the mean absolute inter-nodal error decreasing from 4.3 to 17.1 ppb before calibration to 3.2 to 6.2 ppb after calibration (non-linear regressions did not result in a major difference). However, the calibration coefficients changed significantly over time due to sensor aging (~6 weeks) [19,26] and following episodic events such as rain and dust storms (within about a day) [19]. It is noteworthy that the OPC sensors were more reliable than the sensors for the gaseous pollutants, with their calibration being more stable over time than that of the EC and MO sensors.

Calibration can be done also in a qualitative manner if the sensors do not report correct values but the measurement errors obey a few rather general assumptions, e.g., additive errors with a zero mean. In such cases, a group-decision-making approach for sensor calibration has been demonstrated for collocated sensors [37]. Briefly, given a set of collocated MSUs, each measurement (pollutant and sensor technology specific) is regarded as a referee’s evaluation. At any time point, the consensus value is obtained by minimizing the sum of the relative measurement errors of all the available observations with respect to it.

In any case, after any calibration, the sensors’ performance tends to decline, resulting in a non-linear inter-nodal (i.e., network) divergence of the MSU observations. This calls for frequent calibrations. However, performing frequent collocations of numerous WDESN nodes at far fewer AQM stations is impractical and will cause a severe loss of measurements at the deployment sites. Moreover, since we have shown that the sensors’ performance is affected by their micro-environmental conditions (Section 3.3 and Section 3.4), calibration while in collocation at an AQM station may not provide the desired sensor performance at the deployment site. Hence, an in situ calibration procedure was sought.

**Figure 1:**
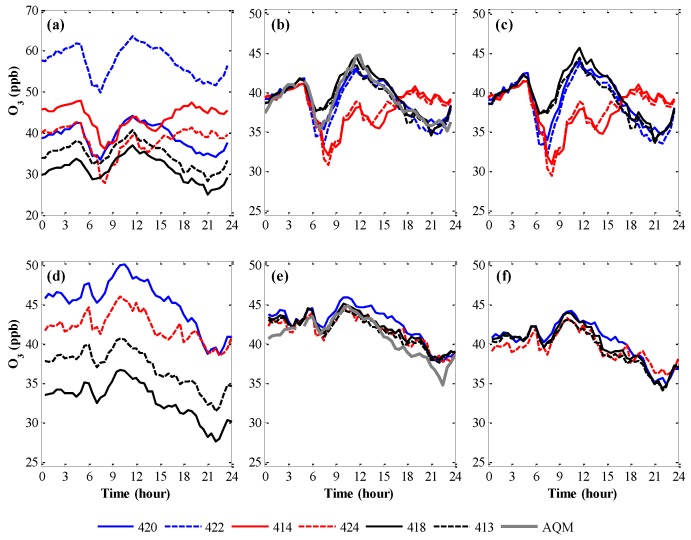
Daily patterns of 30 min. average O_3_ concentrations during weekdays (Sunday to Thursday; upper row) and Saturdays (lower row) (**a**,**d**) before calibration; (**b**,**e**) after calibration against a nearby AQM measurements from 1:00 to 4:00 am; (**c**,**f**) and after calibration against the mean half-hourly reading between 1:00 and 4:00 am of all the WDESN nodes. (Reproduced with permission from [19]).

First, we attempted the in situ calibration of MSUs that were deployed in a residential neighborhood in which an AQM station was present but with which the nodes were not collocated. The MSU observations from 01:00 to 04:00 am were used to calibrate the O_3_ sensors against data collected by the AQM station (~600 to 800 m apart from the deployed WDESN) [19]. Spatially uniform neighborhood-wise O_3_ concentrations were assumed during each time-point within this time-window (30 min intervals), since the local anthropogenic emissions of O_3_ precursors (NO_2_ and volatile organic compounds) were negligible due to marginal traffic and as O_3_ formation did not take place (nighttime atmospheric chemistry). Namely, local ozone production (due to photochemical reactions) and depletion (due to titration with fresh NO) could be ignored. Indeed, based on data from five AQM stations distributed across the Haifa urban residential area, O_3_ concentrations in this time window were relatively homogeneous. Sensor-specific linear regression coefficients were used for adjusting the sensors’ raw O_3_ readings. The calibration of each SMU was evaluated against data collected when the SMUs were re-collocated at the AQM station. The in situ calibration approach reduced the average mean absolute deviation across all the WDESN O_3_ sensors (while collocated) from 13.3 (3.8 to 31.0) ppb before calibration to 1.3 (0.6 to 3.1) ppb after the in situ calibration. For example, the O_3_ daily patterns at three sites during weekdays (Sunday to Thursday) and weekends (Saturday) before and after the in situ calibration are shown in Figure 1. The daily patterns (Figure 1b,e) indicate that the in situ nighttime calibration overcame the disparity among the O_3_ sensor measurements (Figure 1a,d), bringing them to a common ground at night while still revealing spatial variability during the day, with the latter resulting from the spatial variability of weekday traffic related emissions in the neighborhood.

The proposed in situ calibration approach has several limitations, with the major one being that most urban neighborhoods do not have an AQM station within their boundaries; thus, local reference AQM data are unavailable. In such cases, rather than using AQM data, we examined the area using as a reference level the 01:00 to 04:00 am half-hourly mean sensor readings from all the nodes that were deployed in the neighborhood [19]. Whereas this procedure does not assure the calibration of the sensors to the true pollutant concentration, it does bring all the sensors to a common neighborhood-scale baseline and can therefore reveal the relative spatial variability. Namely, this is a conceptual extension of the method proposed in [37] for in situ calibration. We demonstrated this procedure using the same O_3_ data and captured the neighborhood-scale spatiotemporal O_3_ daily patterns (Figure 1c,f) while reducing the average mean absolute deviations among sensors during the re-collocation evaluation phase from 13.3 (3.8 to 31.0) ppb (uncalibrated sensor measurements) to 1.5 (0.7 to 3.7 ppb). The latter turned out to be almost identical to the direct calibration against the AQM data (see above). The theoretical derivation of this calibration procedure [19] proved that the relative ranking of the sensor readings (i.e., the SET match score, [20]) is not disrupted. Hence, as long as neighborhood-scale concentration homogeneity is a valid assumption during the calibration period, calibration against the sensors’ mean observation provides reliable inner-neighborhood spatial patterns. It is noteworthy that the spatial O_3_ concentration patterns were almost identical whether the calibration was done against the AQM data or against the mean observation of all the reporting sensors at any time point (Figure 1).

Since these calibration procedures are done in situ while the MSUs are deployed and reporting, it can be repeated on a daily basis, circumventing the effects of sensor aging and degradation. In fact, it is also possible to apply these calibration procedures in a ‘predictive mode’, with the calibration coefficients for each day calculated based on the pertinent data from previous days. We found that, for the MO O_3_ sensors, data from two to four nights were required for the coefficient of determination (R^2^) to stabilize. Longer periods are undesired as they tend to smooth temporal variations that are characterized by typical time constants smaller than the synoptic time scale [38]. The ‘rolling forwards’ calibration, based on 1:00 to 4:00 am pollutant records from the three preceding nights reduced the average mean absolute deviation between measurements of the O_3_ sensors from 3.7 to 18.7 ppb (before calibration) to 0.5 to 1.1 ppb (during the re-collocation evaluation phase) while still revealing the spatial variability of the O_3_ daily patterns.

The in situ calibration procedures discussed above share a key limitation; they require spatially homogeneous pollutant concentrations during the calibration window. In general, this assumption fits better secondary pollutants (e.g., pollutants formed from precursor emissions rather than directly emitted to the atmosphere) like O_3_ or pollutants with a major contribution from long-range transport like fine PM in Israel. Recently, another approach for in situ field calibration has been proposed [39], which is suitable for the calibration of MSU sensors of primary pollutants, i.e., pollutants that do vary in space and time. Briefly, we examined a node-to-node (N2N) calibration procedure, with only one sensor in each calibration chain directly calibrated against the reference measurements, while the rest of the sensors were calibrated sequentially one against the other while they were deployed in pairs solely during the calibration. The calibration sequence can be applied multiple times. This procedure minimizes the total number of sensor relocations and enables calibration while simultaneously performing measurements at the deployment sites. The N2N calibration procedure was shown to be generic, i.e., applicable for different pollutants, sensing technologies, sensor platforms, chain lengths, and MSU orders within the chain. Our results suggest that the length of the sensor sequence that can be applied for N2N calibration strongly depends on the performance of individual sensors, as well as on the ambient concentrations. Namely, the higher the ambient concentrations, the more accurate are the sensors and the longer the chain that can be applied for N2N calibration, while the accumulated calibration errors remain manageable. Hence, WDESN for air quality measurements are expected to perform better in traffic-affected inner-city sites and in more polluted geographical regions and megapolises. The N2N calibration of individual sensors was found to be comparable to direct calibration by means of collocation at an AQM station, but the flexibility of N2N calibration enables more frequent sensor calibrations.

## 4. Discussion

**Figure 2:**
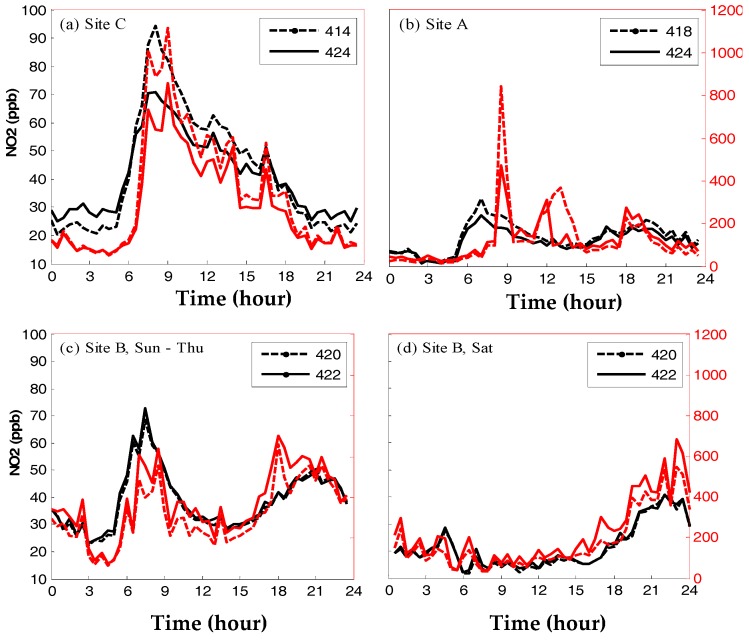
Daily patterns of 30 min average NO_2_ (black) and total volatile organic compounds (TVOC) (red) concentrations in (**a**) site C during weekdays (Sunday to Thursday); (**b**) site A during weekdays (note the adaptation of node 424 to its microenvironments upon relocation); (**c**) site B during weekdays; and (**d**) site B during Saturdays. The MSUs were deployed in a residential neighborhood after they were calibrated while collocated at an AQM station within the neighborhood. (Reproduced with permission from [19]).

Whereas off-the-shelf WDESN MSUs for air pollution measurement are available, in most cases they have not been evaluated rigorously to ensure adequate performance prior to marketing. Currently, to the best of our knowledge, all the available MSUs do not meet the Air Quality Directive 2008/50/EC [40] criteria for regulatory purposes since their uncertainty does not meet the data quality objectives [27]. In this study, we highlighted the potential and the challenges of WDESN technology, which are related to the diverse environmental conditions under which these relatively-cheap MSUs are expected to operate and report reliable observations: varied microenvironments, varying meteorological and environmental conditions, continuous sensor degradation, and general malfunction. We presented different calibration procedures, asserting the need for frequent in situ calibration. We also presented methods to relax the requirements from a calibration procedure when reference measurements are not available, which may fit applications in which precision is important but accuracy can be relaxed and when the data quality does not need to comply with the standards required by regulations or for research [21]. We showed that the SET match score [20] provides quantitative information on whether the sensors are precise, i.e., can capture air pollution patterns. In particular, we found that our EC NO sensors (match score of ~0.8) and the OPC-transformed PM_10_ records (match score of ~0.9) consistently reported reliable air quality patterns [27], whereas other EC sensors (NO_2_, CO, O_3_) and the PM_2.5_ data showed unsatisfactory match scores (<0.5). In the future, progress in sensor technology will probably result in WDESNs that reliably measure different pollutants. Meanwhile the current state-of-the-art WDESNs that measure gaseous pollutants mostly fit applications that do not require high data reliability such as raising the public’s awareness of air pollution, empowering citizens to reduce their personal exposure to air pollution, citizen science, and school field-lab demonstrations. As an example, Figure 2 depicts distinguishable spatial variability at the neighborhood scale, as captured by MO WDESN nodes that were deployed (in pairs) in three sites ~100 to 150 m apart [19], with the spatial redundancy (i.e., two MSUs at each site) used for evaluation purposes.

## 5. Conclusions

The new WDESN promise for routine air quality measurement has huge potential for providing intra-urban information on concentrations of airborne pollutants at an unprecedented spatiotemporal resolution. As such, they are expected to capture the dynamic spatial variability at a very high spatiotemporal resolution and (if proved reliable) to become the method of choice for exposure assessment, enabling true tracking of the individual’s trajectory across different microenvironments throughout the day (i.e., accounting for personal time-location-activity).

We demonstrated MSU evaluation by a range of criteria and provided a rich assessment of their performances under varied conditions. Due to the continuous degradation of the sensors’ performance, as well as due to the varying conditions of the microenvironments where the MSUs are deployed, frequent calibrations (or at least evaluations) of the WDESN nodes are required. We presented various methods for in situ field evaluation/calibration, demonstrating how they can pinpoint systematic errors and sensor-specific malfunctions. In general, the high performance statistics reported for WDESN sensors when they are tested in the lab are misleading since they represent unrealistic conditions in which the temperature, relative humidity, and pollutant concentrations are all constant for sufficiently long periods. In particular, our results show that good performance in the laboratory is not indicative of an acceptable performance under real-world conditions, neither for absolute nor for relative values. Moreover, due to the relatively high quantification limit of common low-cost sensors for gaseous pollutants, they perform better at highly polluted areas or time-periods. This conclusion holds also for PNC, yet regulators are more interested in PM since currently there are standards for PM but not for PNC. Since PM is less sensitive to sub-micron particles, which are commonly emitted from combustion processes or formed in the atmosphere following reactions among gaseous precursors, MSUs that report PM will provide more reliable data when placed away from busy city centers, i.e., in areas where airborne particles are expected to be larger due to aging processes (e.g., coagulation, hygroscopic growth, etc.). However, since WDESN sensors tend to be more accurate at higher ambient concentrations, when applying in situ N2N calibration for gaseous sensors, longer MSU chains are expected to be feasible at the city center.

The main challenge in using commercial WDESNs is related to the sensors’ robustness and measurement repeatability. It has been shown that it is necessary to perform frequent in situ field calibration for each sensor individually. Currently, the considerable sensitivity of the sensors to varying environmental conditions makes them unsuitable for air quality legislative compliance applications or for other applications that require high accuracy. However, some MSU sensors were found to be precise and thus capable of providing coarser information on air quality, which could be suitable to specific applications such as raising the awareness or engaging the community. We showed that, after rigorous quality assurance, WDESNs can reveal inner-city spatiotemporal patterns of ambient pollutants. A periodical in situ calibration was shown to reduce the inter-nodal measurement error among collocated nodes and to address the effects of aging and general sensor degradation. As the calibration of WDESNs is a major obstacle to their widespread deployment, an in situ calibration scheme, such as N2N field calibration [39], may enable such frequent calibrations during the deployment of MSUs and require manageable efforts. Clearly, future improvements in sensor technology may reduce the need for such a practice.

## Figures and Tables

**Table 1:** Specifications of the stationary Monitoring Sensor Units (MSUs) for gaseous pollutant measurement.

Pollutant	CO	NO	NO_2_	O_3_	NO_2_	O_3_	Total VOC (TVOC) (CO_2_-Equivalent)
Sensor technology	Electrochemical (EC)	Electrochemical (EC)	Electrochemical (EC)	Electrochemical (EC)	Metal oxide (MO)	Metal oxide (MO)	Metal oxide (MO)
Sensor provider	Alphasense	Alphasense	Alphasense	Alphasense	Applied Sensors	Aeroqual	Applied Sensors
Sensor type	CO-B4	NO-B4	NO2-B42F	OX-B421	iAQ-100	SM50	iAQ-100
Platform manufacturer	Environmental Instruments (UK)	Environmental Instruments (UK)	Environmental Instruments (UK)	Environmental Instruments (UK)	Perkin Elmer (USA)	Perkin Elmer (USA)	Perkin Elmer (USA)
MSU type	AQMesh	AQMesh	AQMesh	AQMesh	Elm	Elm	Elm
Measurement range Sampling frequency	0–5000 ppb 1 min	0–2000 ppb 1 min	0–200 ppb 1 min	0–200 ppb 1 min	10–2000 ppb 20 s	0–150 ppb 1 min	0–2000 ppm 20 s

**Table 2:** Specifications of the stationary MSUs for particle number concentration (PNC) measurement.

MSU	AQMesh (v3.5)	Dylos (1700 DC)
Method	Light scattering	Light scattering
Particle size range	0.3 µm–30 µm	0.5 µm–20 µm
Number of size channels	32	2
Flow rate	0.5 lit/min	1.0 lit/min
Max concentration	2 × 10^6^/lit	3.5 × 10^4^/lit
Platform manufacturer	Environmental Instruments (UK)	Dylos (USA)

**Table 3:** Specifications of the personal MSUs for pollutant measurements.

Pollutant	NO	NO_2_	O_3_	PNC
Sensor technology	Electrochemical (EC)	Electrochemical (EC)	Electrochemical (EC)	Light scattering
Sensor provider	Alphasense	Alphasense	Alphasense	Tzoa
Sensor type	NO-A4	NO2-A42F	OX-A421	OPC
MSU type	LEO	LEO	LEO	Tzoa-RD
MSU manufacturer	ATEKNEA (ES)	ATEKNEA (ES)	ATEKNEA (ES)	Tzoa (CA)
Sampling rate	10 s	10 s	10 s	1 min

**Table 4:** Reference instruments used for the laboratory performance evaluation.

Instrument	Model	Method	Detection Limit
CO analyzer	Teledyne API 300E	Non-dispersive IR spectroscopy (EN14626)	40 ppb
NO_x_ analyzer	Teledyne API 200A	Chemiluminescence (EN 14211)	0.4 ppb
O_3_ analyzer	Teledyne API 400	UV photometry (EN14625)	0.4 ppb

**Table 5:** Performance of the EC sensor in the lab (*n* = 3). In parenthesis are the simultaneous reference measurements.

Platform	Averaging Time (min)	Pollutant	Mean ± STD at Zero-Air (ppb)	Mean ± STD at 100 ppb span * (ppb)	R^2^	Gain	Intercept (ppb)	Cross-Sensitivity
AQMesh	15	CO	16.3 ± 6 (1.9 ± 0.7)	1292 ± 21.5 (1385 ± 16.2)	0.99	0.86	0.07	NO_2_
		NO	n/a (0.4 ± 0.4)	88.5 ± 1.5 (94.1 ± 0.9)	0.99	0.97	−1.13	
		NO_2_	n/a (0.7 ± 0.3)	126.4 ± 3.5 (103.9 ± 0.7)	0.99	1.22	−1.02	
		O_3_	n/a (0.8 ± 0.2)	123.4 ± 2.3 (108.5 ± 1.5)	0.99	1.16	−1.27	
LEO	1	NO_2_	15.3 ± 10.8 (0.4 ± 0.3)	49.0 ± 8.7 (94.3 ± 0.6)	0.99	0.86	23.9	NO_2_
		NO	24.7 ± 3.1 (0.3 ± 0.2)	117.9 ± 3.3 (107.7 ± 0.4)	0.99	0.71	−21.5	
		O_3_	6.8 ± 4.1 (0.5 ± 0.5)	57.5 ± 3.4 (86.1 ± 0.6)	0.96	0.70	−7.7	

* Except for CO, where 1300 ppb was used as the span value.

**Table 6:** Sample Sensor Evaluation Toolkit (SET) analysis. IPI (Integrated Performance Index) of the MSU sensors for ambient nitrogen oxide (NO), nitrogen dioxide (NO_2_), ozone (O_3_), and carbon monoxide (CO). Color code: red (<0.50), magenta (0.50–0.59), yellow (0.60–0.69), light blue (0.70–0.79), dark blue (0.80–0.89), green (0.90–1.00).

MSU #	Deployment	NO	NO_2_	O_3_	CO
001	Barcelona, Spain 2–9 July 2014	0.66	0.46	0.69	0.59
002		0.41	0.46	0.67	0.57
003		0.56	0.50	0.70	0.58
004		0.67	0.50	0.68	0.57
005		0.64	0.48	0.67	0.58
221	Belgrade, Serbia	0.79	0.59	0.59	0.69
222	15 January–27 May 2014	0.79	0.55	0.54	0.69
116	Edinburgh, Scotland 25 March–7 July 2014	0.51	0.41	0.65	0.52
118		0.50	0.39	0.60	0.45
120		0.51	0.41	0.63	0.48
135	Haifa, Israel 16 December 2013–27 April 2014	0.51	0.62	0.66	0.56
136		0.51	0.64	0.70	0.55
130		0.62	n.a.	0.64	n.a.
134		0.63	n.a.	0.63	n.a.
125	Ljubljana, Slovenia 25 February–5 June 2014	n.a.	0.55	0.71	0.77
128		n.a.	0.46	0.69	0.68
131		n.a.	0.54	0.72	0.66
124	Oslo, Norway 13 February–2 June 2014; 25 June–3 September 2014; 15 September–9 December 2014	0.92	0.69	n.a.	0.71
144		0.87	0.64	n.a.	0.70
145		0.86	0.71	n.a.	0.70
146		0.90	0.70	n.a.	0.68
147		0.88	0.57	n.a.	0.59
611	Ostrava, Czech Rep. 19 December 2014–15 January 2015	n.a.	n.a.	0.74	n.a.
612		n.a.	n.a.	0.77	n.a.
143	Vienna 1 January–8 August 2014	0.39	0.35	0.40	n.a.

n.a.—Reference data were unavailable for the analysis.

**Table 7:** Selected SET results for PM_10_ measurements in Ostrava (CZ). Sampling period: 1 June to 7 September 2015, sampling rate: 1 h, color code as in Table 6.

		No. of Data Points (Presence, %)	PM_10_ Mean (STD) (μg/m^3^)	Match Score	Pearson Corr.	Spearman Corr.	Kendall Corr.	LFE	IPI
AQM		2327 (98)	28.78 (16.97)						
MSU no.	693	2375 (100)	10.91 (11.10)	0.31	0.39	0.67	0.50	0.89	0.68
	734	2256 (95)	18.23 (12.10)	0.42	0.54	0.62	0.46	0.98	0.71
	745	1771 (75)	19.94 (10.50)	0.63	0.60	0.64	0.47	0.99	0.72
	749	2373 (~100)	17.03 (9.69)	0.58	0.65	0.68	0.51	0.99	0.77
	788	2372 (~100)	8.50 (5.56)	0.40	0.50	0.61	0.46	0.98	0.71
	813	2374 (~100)	8.71 (6.08)	0.38	0.58	0.66	0.49	0.97	0.72

**Table 8:** Average node-to-node and node-to-air quality monitoring (AQM) correlations using collected personal Little Environmental Observatory (LEO) nodes data. Each experiment lasted 4 h (sampling rate 1 min). Study area: Haifa, Israel.

Scenario		NO_2_	O_3_	NO
Experiment 1 (5.11.15)	Average inter-MSU correlation	0.98	0.62	0.53
	Average MSU-AQM correlation	0.80	0.58	0.50
Experiment 2 (19.11.15)	Average inter-MSU correlation	0.75	0.40	0.50
	Average MSU-AQM correlation	0.11	0.05	0.50
Experiment 3 (26.11.15)	Average inter-MSU correlation	0.86	0.38	0.69
	Average MSU-AQM correlation	0.76	0.10	0.71

## References

[1] Carminati M., Ferrari G., Sampietro M. (2017). Emerging miniaturized technologies for airborne particulate matter pervasive monitoring. Measurement.

[2] Hasenfratz D., Saukh O., Walser C., Hueglin C., Fierz M., Arn T., Beutel J., Thiele L. (2015). Deriving high-resolution urban air pollution maps using mobile sensor nodes. Pervasive Mob. Comput..

[3] Broday D.M., Carmel Y. (2005). Mapping spatiotemporal variables: The impact of the time-averaging window width on the spatial resolution. Atmos. Environ..

[4] Broday D.M. (2006). High resolution spatial patterns of long-term mean air pollutants concentrations in Haifa Bay area. Atmos. Environ..

[5] Levy I., Levin N., Schwartz J.D., Kark J.D. (2015). Back-extrapolating a land use regression model for estimating past exposures to traffic-related air pollution. Environ. Sci. Technol..

[6] Bekhor S., Broday D.M. (2013). Data-driven nonlinear optimization of a simple air pollution dispersion model generating high resolution spatiotemporal exposure. Atmos. Environ..

[7] Chen S., Bekhor S., Broday D.M. (2016). Aggregated GPS tracking of vehicles and its use as a proxy of traffic-related air pollution emissions. Atmos. Environ..

[8] Kanaroglou P., Jerrett M., Morrison J., Bernardo Beckerman M., Arain A., Gilbert N., Brook J. (2005). Establishing an air pollution monitoring network for intra-urban population exposure assessment: A location-allocation approach. Atmos. Environ..

[9] Stetter J.R., Li J. (2008). Amperometric gas sensors—A review‏. Chem. Rev..

[10] Bard A., Faulkner L. (2001). Electrochemical Methods: Fundamentals and Application.

[11] Gibson D., MacGregor C. (2013). A novel solid state non-dispersive Infrared CO_2_ gas sensor compatible with wireless and portable deployment. Sensors.

[12] Carminati M., Pedalàa L., Bianchi E., Nasonb F., Dubini G., Cortelezzi L. (2014). Capacitive detection of micrometric airborne particulate matter for solid-state personal air quality monitors. Sens. Actuators A.

[13] Gao R.S., Telg H., McLaughlin R.J., Ciciora S.J., Watts L.A., Richardson M.S., Schwarz J.P., Perring A.E., Thornberry T.D., Rollins A.W. (2016). A light-weight, high-sensitivity particle spectrometer for PM_2.5_ aerosol measurements‏. Aerosol Sci. Technol..

[14] Manikonda A., Zíková N., Hopke P.K., Ferro A.R. (2016). Laboratory assessment of low-cost PM monitors. J. Aerosol Sci..

[15] Yu C.H., Fan Z.-H., Lioy P.J., Baptista A., Greenberg M., Laumbach R.A. (2016). Novel mobile monitoring approach to characterize spatial and temporal variation in traffic-related air pollutants in an urban community. Atmos. Environ..

[16] Mead M., Popoola O., Stewart G., Landshoff P., Calleja M., Hayes M., Baldovi J., McLeod M., Hodgson T., Dicks J. (2013). The use of electrochemical sensors for monitoring urban air quality in low-cost, high-density networks. Atmos. Environ..

[17] Lerner U., Yacobi T., Levy I., Moltchanov S., Cole-Hunter T., Fishbain B. (2015). The effect of ego-motion on environmental monitoring. Sci. Total Environ..

[18] Cai J., Yan B., Kinney P.L., Perzanowski M.S., Jung K.-H., Li T., Xiu G., Zhang D., Oliv C., Ross J. (2013). Optimization approaches to ameliorate humidity and vibration related issues using the microAeth black carbon monitor for personal exposure measurement. Aerosol Sci. Technol..

[19] Moltchanov S., Levy I., Etzion Y., Lerner U., Broday D.M., Fishbain B. (2015). On the feasibility of measuring air pollution at dense urban areas by wireless distributed sensor networks. Sci. Total Environ..

[20] Fishbain B., Lerner U., Cole-Hunter T., Castell-Balaguer N., Popoola O., Broday D., Martinez-Iñiguez T., Nieuwenhuijsen M., Jovasevic-Stojanovic M., Topalovic D. (2017). An evaluation tool kit of air quality micro-sensing units. Sci. Total Environ..

[21] Snyder E.G., Watkins T.H., Solomon P.A., Thoma E.D., Williams R.W., Hagler G.S.W., Shelow D., Hindin D.A., Kilaru V.J., Preuss P.W. (2013). The changing paradigm of air pollution monitoring. Environ. Sci. Technol..

[22] Al Ali A., Zualkernan I., Aloul F. (2010). A mobile GPRS-sensors array for air pollution monitoring. IEEE Sens. J..

[23] Levy I., Mihele C., Lu G., Narayan J., Hilker N., Brook J. (2014). Elucidating multipollutant exposure across a complex metropolitan area by systematic deployment of a mobile laboratory. Atmos. Chem. Phys..

[24] Honicky R. (2011). Towards a Societal Scale, Mobile Sensing System. Ph.D. Thesis.

[25] Wang C., Yin L., Zhang L., Xiang D., Gao R. (2010). Metal oxide gas sensors: Sensitivity and influencing factors. IEEE Sens. J..

[26] Williams D., Henshaw G., Bart M., Laing G., Wagner J., Naisbitt S., Salmond J.A. (2013). Validation of low-cost ozone measurement instruments suitable for use in an air-quality monitoring network. Meas. Sci. Technol..

[27] Castell N., Dauge F.R., Schneider P., Vogt M., Lerner U., Fishbain B., Broday D., Bartonova A. (2017). Can commercial low-cost sensor platforms contribute to air quality monitoring and health exposure estimates?. Environ. Int..

[28] Lin C., Gillespie J., Schuder M.D., Duberstein W., Beverland I.J., Heal M.R. (2015). Evaluation and calibration of Aeroqual series 500 portable gas sensors for accurate measurement of ambient ozone and nitrogen dioxide. Atmos. Environ..

[29] Zikova N., Masiol M., Chalupa D.C., Rich D.Q., Ferro A.R., Hopke P.K. (2017). Estimating hourly concentrations of PM_2.5_ across a metropolitan area using low-cost particle monitors. Sensors.

[30] Seinfeld J.H., Pandis S.N. (2016). Atmospheric Chemistry and Physics: From Air Pollution to Climate Change.

[31] Spinelle L., Gerboles M., Villani M., Aleixandre M., Bonavitacola F. (2017). Field calibration of a cluster of low-cost commercially available sensors for air quality monitoring. Part B: NO, CO and CO_2_. Sens. Actuators B.

[32] Cyrys J., Pitz M., Heinrich J., Wichmann H.R., Peters A. (2008). Spatial and temporal variation of particle number concentration in Augsburg, Germany. Sci. Total Environ..

[33] Etzion Y., Broday D.M. Highly resolved spatiotemporal variability of fine particle concentrations in an urban neighborhood. Proceedings of the European Aerosol Conference.

[34] Broday D.M., Rosenzweig R. (2011). Deposition of fractal-like soot aggregates in the human respiratory tract. J. Aerosol Sci..

[35] Heinzerling A., Hsu J., Yip F. (2016). Respiratory health effects of ultrafine particles in children: A literature review. Water Air Soil Pollut..

[36] Wang F., Ketzel M., Ellermann T., Wahlin P., Jensen S.S., Fang D., Massling A. (2010). Particle number, particle mass and NOx emission factors at a highway and an urban street in Copenhagen. Atmos. Chem. Phys..

[37] Fishbain B., Moreno-Centeno E. (2016). Self calibrated wireless distributed environmental sensory networks. Sci. Rep..

[38] Broday D.M. (2010). Studying the time scale dependence of environmental variables predictability using fractal analysis. Environ. Sci. Technol..

[39] Kizel F., Etzion Y., Shafran-Nathan R., Levy I., Fishbain B., Bartonova A., Broday D.M. (2017). Node-to-node field calibration of wireless distributed air pollution sensor network. Environ. Pol..

[40] European Union (EU) (2008). Directive 2008/50/EC of the European Parliament on Ambient Air Quality and Cleaner Air for Europe.

